# Concurrent Oncolysis and Neurolesion Repair by Dual Gene-Engineered hNSCs in an Experimental Model of Intraspinal Cord Glioblastoma

**DOI:** 10.3390/cells13181522

**Published:** 2024-09-11

**Authors:** Xiang Zeng, Alexander E. Ropper, Zaid Aljuboori, Dou Yu, Theodore W. Teng, Serdar Kabatas, Esteban Usuga, Jamie E. Anderson, Yang D. Teng

**Affiliations:** 1 Department of Physical Medicine and Rehabilitation, Harvard Medical School and Spaulding Rehabilitation Hospital, Boston, MA 02129, USA; 2 Department of Neurosurgery, Harvard Medical School and Brigham and Women’s Hospital, Boston, MA 02115, USA; 3 Laboratory of SCI, Stem Cell, and Recovery Neurobiology Research, Department of Physical Medicine and Rehabilitation, Spaulding Rehabilitation Hospital Network, Mass General Brigham, and Harvard Medical School, Boston, MA 02129, USA; 4 Harvard College, Harvard University, Cambridge, MA 02138, USA

**Keywords:** intramedullary spinal cord tumor, glioblastoma, neural stem cells, gene-directed enzyme prodrug therapy/GDEPT, locomotion, autonomic dysfunction

## Abstract

Intramedullary spinal cord glioblastoma (ISCG) is lethal due to lack of effective treatment. We previously established a rat C6-ISCG model and the antitumor effect of F3.CD-TK, an hNSC line expressing CD and TK, via producing cytocidal 5FU and GCV-TP. However, the neurotherapeutic potential of this hNSC approach has remained uninvestigated. Here for the first time, cultured F3.CD-TK cells were found to have a markedly higher oncolytic effect, which was GJIC-dependent, and BDNF expression but less VEGF secretion than F3.CD. In Rowett athymic rats, F3.CD-TK (1.5 × 10^6^ cells/10 µL × 2), injected near C6-ISCG (G55 seeding 7 days earlier: 10 K/each) and followed by q.d. (×5/each repeat; i.p.) of 5FC (500 mg/kg/5 mL/day) and GCV (25 mg/kg/1 mL/day), robustly mitigated cardiorespiratory, locomotor, and sensory deficits to improve neurofunction and overall survival compared to animals receiving either F3.CD or F3.CD-TK+F3.CD debris formula. The F3.CD-TK regimen exerted greater tumor penetration and neural inflammation/immune modulation, reshaped C6-ISCG topology to increase the tumor’s surface area/volume ratio to spare/repair host axons (e.g., vGlut1+ neurites), and had higher post-prodrug donor self-clearance. The multimodal data and mechanistic leads from this proof-of-principle study suggest that the overall stronger anti-ISCG benefit of our hNSC-based GDEPT is derived from its concurrent oncolytic and neurotherapeutic effects.

## 1. Introduction

Intramedullary spinal cord glioblastoma (ISCG) remains a lethal disease due to its poor response to conventional interventions [1,2,3]. Marked by its initial nonspecific symptoms, ISCG can swiftly worsen neurological function later because ~60% of cases involve the cervical or cervicothoracic cord, which regulates essential autonomic and somatic functions [4]. The migratory, metastatic, and diffuse growth profile of ISCG and the delicate anatomy of the spinal cord render aggressive surgical treatment insufficient and challenging [2,4]. Unfortunately, ISCG has to date been given little attention in research, and development of efficacious therapies remains an urgent clinical demand [3,4].

We earlier established a rat xenograft model of C6-ISCG and demonstrated that F3.CD-TK, dual gene-engineered human neural stem cells (hNSCs) expressing cytosine deaminase (CD) and herpes simplex virus thymidine kinase (TK), could migrate toward human ISCG cells led by their inherent tumoritropic property, a key trait of NSC functional multipotency [5,6]. The F3.hNSC-produced CD and TK converted nontoxic 5-fluorocytosine (5FC) and ganciclovir (GCV) into oncolytic 5-fluorouracil (5FU) and GCV-triphosphate (GCV-TP), respectively, to eliminate cancer cells [5,6,7]. These capacities enabled F3.CD-TK cells to exert bystander oncolytic effects to suppress xenograft C6-ISCG and commit suicidal self-clearance [5,6]. Of note is that F3.CD-TK is a variant line of the HB1.F3.CD (also termed F3.CD: producing CD only) that, following laboratory safety and efficacy confirmation [8,9], has been used in clinical studies to treat reoccurring brain glioblastoma multiforme (GBM) [10,11].

Managing tumor cells is only one key aspect of an anti-ISCG therapy since both glioblastoma (GB) growth and established anticancer therapies cause debilitating neuroinjuries [2,3,4]. Given the ability of NSCs to promote neural recovery [12,13], it is crucial to investigate whether the general benefit of the F3.hNSCs is produced by their ability to concurrently kill cancer cells and mitigate tumor-triggered neurolesions. Based on the mechanisms of the functional multipotency of stem cells and recovery neurobiology of injured spinal cords [12,13], we hypothesized that F3.CD-TK treatment might not only suppress ISCG growth but also offer means of neurorepair. To test this, systematically designed assays were conducted in vitro and in C6-ISCG rats, the only cervical rodent model available, to multimodally determine the oncolytic and neuroprotective effects of the F3.CD-TK regimen, compare the efficacy and donor self-clearance differences between F3.CD-TK and F3.CD treatments, and uncover mechanistic leads.

## 2. Materials and Methods

### 2.1. Cell Culture

Human glioblastoma cell line G55: This line was initially developed by Dr. C. David James of UCSF by passaging human GBM cells through nude mice to reestablish a stable patient-derived-xenograft GBM cell line [14]. Dr. Evan Y. Snyder of Sanford Burnham Prebys Medical Discovery Institute kindly provided the G55 cell line for this study [6]. Notably, G55 cells are isocitrate dehydrogenase (IDH) 1 and 2 wildtype [15] and have other signature genes, plus a VEGF profile of glioblastoma in the family of adult-type diffuse gliomas based on the 2021 WHO classification of tumors of the CNS [16,17,18]. G55 cells have also been found to be more invasive than other glioma cell lines, such as U87 [6,19]. Moreover, the G55 GBM model, characterized by a heightened vascularity and necrosis propensity, exhibits more characteristics of clinical primary GB [17,19].

Per our published protocols [5,6], G55 cells were maintained (37 °C and 5% CO_2_) with Dulbecco’s modified Eagle’s medium (DMEM) (Life Technologies, Grand Island, NY, USA) with 10% fetal bovine serum (FBS) (Atlanta Biologicals, Flowery Branch, GA, USA) and 1% penicillin and streptomycin (Life Technologies). Cells were split when reaching ~80% confluency with application of 0.25% trypsin (Life Technologies).

F3.CD and F3.CD-TK hNSC lines: We received both cell lines from Dr. Seung U. Kim and Dr. Hong Jun Lee of University of British Columbia, Vancouver, BC, Canada and Chung-Ang University, Seoul, Korea as a research collaboration gift [5]. F3.CD and F3.CD-TK cells were genetically engineered hNSCs derived from the parental F3 hNSC line [7,20,21]. Briefly, expression plasmids encoding Escherichia coli CD (1.5 KB) and CD-TK (2.53 KB) were made using the retroviral pLPCX backbone and CD and CD-TK cDNA. Vector packaging was done by co-transfection of pA317 cells with the CD or CD-TK puro plasmid and the MV12 envelope-coding plasmid. The CD or CD-TK puro retroviral supernatant was applied to infect F3 hNSC cells, followed with selection (3 mg/mL puromycin) to attain transduced cells (purity: 100%). In this study, F3.CD and F3.CD-TK cells were cultured according to the same protocol used for G55 (see above).

The dose-response assay of the oncolytic effect: Concisely, under the 1:2 (7500 vs. 15,000 cells/well) optimal ratio of F3.CD or F3.CD-TK and G55 co-culturing of 24 h, a bolus dose of 5FC (MW: 129.1) (Sigma-Aldrich, St. Louis, MO, USA) at a final concentration of 2.1 mM (270 μg/mL) or the same dose of 5FC plus 12 μM (3 μg/mL) GCV (MW: 255.2) (Sigma-Aldrich) was applied to each well (3 × 3 wells/each dose/each assay; total: 9 assays). After 72 h co-culturing, cells were processed for measuring the IRL of activated caspase 3 (see below).

### 2.2. DiI Labeling of F3.CD and F3.CD-TK

DiI prelabeling of F3.hNSCs was performed 48 h before G55 coculture or transplantation by directly adding Cell Tracker CM-DiI (Life Technologies) to the culture medium (final concentration: 2 μM). The cells were incubated (37 °C) in the dye solution for 20 min before rinsing three times with DPBS (Dulbecco’s Phosphate Buffered Saline; Life Technologies) and fresh medium addition. CM-DiI has maximum fluorescence emission at 570 nm, and the DiI-prelabeled F3.hNSCs appeared red under a Zeiss Texas Red filter.

### 2.3. In Vitro Assay for the Tumoricidal Effect of F3.CD and F3.CD-TK Formulas 

The optimal 5FC and/or GCV concentrations for the bystander cytotoxic effect of F3.CD or F3.CD-TK cells on G55 cells were systematically determined as per the protocol described below (see Results for experimental group size specification).

- The dose–response oncolytic effect of 5FC on G55 converted by F3.CD or F3.CD-TK: G55 cells (1.5 × 10^4^/well) and F3.CD or F3.CD-TK cells (0.75 × 10^4^/well) were sequentially seeded in 24-well plates (BD Falcon; BD Biosciences, San Jose, CA, USA). After 24 h, a bolus dose of 5FC (0, 0.7 mM, 2.1 mM, or 7 mM) (Sigma-Aldrich) was added into the culture medium, and the culture was kept for another 72 h (total: 96 h).
- The dose–response effect of 5FC and GCV on G55 cells converted by F3.CD-TK cells: G55 cells (1.5 × 10^4^/well) and F3.CD-TK cells (0.75 × 10^4^/well) were sequentially seeded in 24-well plates (n = 5/group). After 24 h, a combinatorial dose of 5-FC (0, 0.7 mM, 2.1 mM, or 7 mM) and GCV (0, 4 µM, 12 µM, or 40 µM) (Sigma-Aldrich) was applied to the culture medium, and the culture was maintained for another 72 h (total: 96 h).
- Comparison of the oncolytic potency of individually or combinatorially applied 5FC and GCV in F3.CD-TK and G55 cocultures: G55 cells (1.5 × 10^4^ per well) and F3.CD-TK cells (0.75 × 10^4^ per well) were sequentially seeded in 24-well plates. After 24 h, a single dose of 5FC at 2.1 mM or GCV at 12 µM, or a combination of 2.1 mM 5FC + 12 µM GCV (i.e., optimal single doses determined in Steps i and ii), was applied to the culture medium to continue incubation for an additional 72 h.
- Evaluation of the oncolytic effect of consecutive prodrug administration on G55 cells converted by F3.CD-TK cells: G55 cells (1.5 × 10^4^/well) and F3.CD-TK cells (0.75 × 10^4^/well) were sequentially seeded in 24-well plates. After 24 h, 5FC or GCV was administered according to the following formulas: (1) 2.1 mM 5FC for 72 h; (2) 12 µM GCV for 72 h; (3) 2.1 mM 5FC for the first 36 h followed with 12 µM GCV for another 36 h; (4) 2.1 mM 5FC for the first 24 h with subsequent medium change with no precursor drug for 24 h (i.e., a washout period), followed by another 24 h treatment of 12 µM GCV; (5) 12 µM GCV for the first 36 h followed with 2.1 mM 5FC for another 36 h; (6) 12 µM GCV for 24 h with a subsequent 24 h washout period (see above), followed by another 24 h treatment of 2.1 mM 5FC; (7) combinatorial treatment of 2.1 mM 5FC and 12 µM GCV for 72 h; (8) no prodrug culturing for 72 h. At the end of each experiment, cell counting was conducted using the standard trypan blue method [5]. Each independent assay was performed in triplicate.

### 2.4. Immunocytochemical (ICC) Analysis of Activated Caspase 3

The bystander oncolytic effect on G55 cells was reconfirmed by the ICC assay of cleaved caspase-3 as per protocols published previously [5,6]. Briefly, cells in the aforementioned settings of F3.CD and G55 cells under 2.1 mM 5FC or F3.CD-TK + G55 under 2.1 mM 5-FC + 12 µM GCV were fixed with 2% paraformaldehyde (PFA) for 1–2 h before washing with PBS containing 0.1% Triton X-100 (vol/vol) and incubated in 3% (vol/vol) normal donkey serum (Millipore) for 30 min at room temperature (RT). Primary antibody for cleaved caspase-3 (#9661; Cell Signaling Technology, Inc., Danvers, MA, USA) was applied to cells for overnight incubation (4 °C). After washing and secondary antibody incubation, cells were washed in PBS, and the nuclei were counterstained with DAPI (Vector Labs, Newark, CA, USA) for epifluorescence microscope evaluation (Axiovert 200 microscope equipped with an Axiocam CCD camera; Zeiss).

### 2.5. Enzyme-Linked Immunosorbent Assay (ELISA) of VEGF and BDNF

F3.CD, F3.CD-TK, and G55 cells under standard maintenance (see above) were detached, dissociated, and reseeded (3000, 10,000, or 30,000 cells/well) in a 24-well plate. For coculture of F3.CD or F3.CD-TK with G55 cells, 15,000 cells of either cell type were jointly seeded into one well. Cells were incubated in DMEM containing 10% FBS overnight to facilitate attachment. After gentle washing (3× warm DPBS), α-MEM (Sigma-Aldrich) was added for another 24 h culturing. Supernatant from each well was collected and immediately frozen at −80 °C till analyzing. ELISA assay for VEGF and BDNF was carried out according to the manual provided by the manufacturer (Life Technologies, Grand Island, NY, USA). Absorbance of the blue products of HRP-TMB chromogenic reaction was read by Microplate Spectrophotometers (FlexStation 3, Molecular Devices, Sunnyvale, CA, USA) at 450 nm and 550 nm. Each sample was measured three times to obtain a mean value. Lastly, subtraction of 550 nm values from 450 nm values was used to correct for the optical imperfections of the microplate. 

### 2.6. In Vivo Study Design

The effects of genetically engineered F3.hNSCs on ISCG were systematically tested through utilizing a randomized block-design for the in vivo investigation. Cell debris was prepared by processing F3.CD and F3.CD-TK cells (1:1) of the same concentration via ≥3× freeze (−80 °C × 1 h)-thaw (37 °C × 30 min) cycles, with the result being confirmed by the trypan blue exclusion assay [5]. Statistical power analysis was performed based on a set of preliminary data. As reported before [5], with 4 rats per group, there was ~95.5% possibility of detecting ≥42.7% of difference in mean survival time among F3.CD-TK-treated, F3.CD-treated, and F3.CD-TK- and F3.CD-cell-debris-treated groups. It was thereby determined that a group size of 6 would be adequate for analyzing the main outcome measures of this study [5,6,12]. All in vivo procedures were approved by the Harvard Medical Area Standing Committee on Animals.

### 2.7. Intramedullary Spinal Cord Tumor Model

All animals completed the study and were examined behaviorally and neural functionally as per our protocols. Based on our established procedure [5], adult (8–9 weeks-old) immunodeficient female rats (Rowett Nude; body weight: 175–190 g; Charles River Laboratories, Wilmington, MA, USA) were i.p. anesthetized with 75 mg/kg ketamine hydrochloride and 10 mg/kg xylazine (Patterson Veterinary, Devens, MA, USA). With appropriate support and monitoring, a longitudinal incision was made over the cervical region to expose and remove the C6 spinous process and lamina, followed with resection of the ligamentum flavum to expose the C6-7 dura. A 26-gauge needle (O.D.: 460 µm & I.D.: 260 µm) connected to a Hamilton microsyringe was inserted from dorsal surface to enter the spinal cord (depth: 2 mm). The needle was retracted 0.5 mm (final depth: 1.5 mm) before starting the infusion of G55 cells (10^4^/3 µL PBS/each) over 5 min. The needle was kept in situ for another 5 min before being slowly retracted to prevent any backflow of the cells. Afterward, standard wound closure and postcare were provided including preventive nociception management [22].

### 2.8. Administration of F3.hNSCs and Prodrugs

Seven days after G55 GB implantation, animals were anesthetized and prepared as described above. The incision was reopened, and paravertebral muscles were laterally retracted before C5 and C7 laminectomies were performed using a rongeur to visualize the entire tumor mass. DiI-prelabelled F3.CD or F3.CD-TK cells (1.5 × 10^6^/10 μL PBS/each site) were intramedullary injected (depth: 1.5 mm; duration: 15 min plus 5 min needle retainment) into loci 1 mm rostral and 1 mm caudal to the rostral and caudal tumor margins as described before [5]. Wound closure and postcare were provided as previously described. Equivolume solution of DiI-free cell debris, produced from repeated freezing and thaw of F3.CD and F3.CD-TK cells (see Methods), was accordingly administered to the control animals. 

Then, 2 days (~48 h) after F3.hNSC injection, prodrug administration was initiated as per the logistics detailed below: (i) F3.CD formula: 5-FC (500 mg/kg/day, i.p.; note: based on the in vitro data, no GCV was added) was given for 5 consecutive days. Following a 2-day prodrug-free interval, the same treatment was repeated until the rat met the termination criteria (i.e., BBB score ≤ 9; note: our previous data revealed no detectable effect of 25 mg/kg/day GCV administration on F3.CD-injected rats, so no GCV was given to this group) [5]. (ii) F3.CD-TK regimen: i.p. administrations of 5-FC and GCV ((500 mg/kg/day and 25 mg/kg/day) × 5, respectively, for each repeat) were performed according to the same protocol as the F3.CD formula. (iii) Control treatment: for animals receiving equivolume cell debris (see above), 5FC+GCV administrations as per (ii) were given.

### 2.9. Functional Evaluation and Termination Criteria

Somatomotosensory functions: Rats were monitored daily for general conditions that consisted of body weight, facial expression, porphyrin staining, grooming, hair and skin quality, and spontaneous activity [22]. Locomotor, sensory, and autonomic functions were evaluated weekly by two investigators who were blind to the study group assignment. Hindlimb locomotion was tested as per the standardized Basso, Bresnahan, and Beattie (BBB) scale [23]. Forelimb locomotor assessment was conducted based on an adaptation of an open field forelimb functional rating scale (i.e., forelimb motor score) for rats with cervical spinal cord injury (SCI) [24], which ranges from 0 to 18, to define the articular movements, weight support, digit position, paw placement at initial contact, paw orientation during liftoff, and forelimb and hindlimb coordination (Table S1). 

Autonomic functions: (1) Mean arterial blood pressure, heart rate, and body temperature were measured weekly using a non-invasive tail cuff volume pressure recording device (CODA, Kent Scientific, Torrington, CT, USA) as previously reported [5,25]. (2) For evaluating the respiratory function, animals after adequate acclimation training were placed in a whole-body plethysmographic chamber for preoperative baseline respiratory parameter recording. A 20 min acclimation phase in the chamber preceded all subsequent respiratory data acquisition sessions for each rat to ensure that animals were free of stress signs during the evaluation. As described before [6,26,27], respiratory data were attained noninvasively in unanesthetized, conscious, unrestrained, spontaneously breathing rats using a bias flow-ventilated whole-body plethysmograph (WBP) designed for rodents (PLY3213; Buxco Electronics, Wilmington, NC, USA). The Epstein barometric method was used to derive lung ventilation parameters from pressure fluctuations within chambers of fixed volume. Pressure changes in the chamber were captured by a differential pressure transducer, amplified, and integrated by a software program (BiosystemXA2.7.9; Buxco Electronics, Wilmington, NC, USA) into standard parameters. A negative bias flow regulator set to 1.5 L/min guaranteed that fresh room air was steadily supplied into the chambers and that CO_2_ from breaths expired from the animal did not accumulate. Respiratory rate (f), tidal volume (TV), minute ventilation (MV), inspiration time (IT), expiration time (ET), etc., were collected from each animal. 

Termination criteria: Animal overall survival was reported as a primary endpoint. Specifically, survival was defined according to a predetermined cutoff for hindlimb motor dysfunction. In our design, a hindlimb locomotor ability, which was scored ≤9 of the BBB scale (i.e., the possibility of not fully carrying out selfcare), was set as the criteria for humane termination (i.e., mortality). Therefore, the outcome measure was number of days after tumor implantation, which used a neurologic score as a surrogate for tumor burden and disease progression (see Comments by Dr. Daniel J. Hol in Ropper et al., 2016) [5].

### 2.10. Histopathological and Immunohistochemical (IHC) Procedures

Following the approved protocol, animals were euthanatized before tissue perfusion and fixation with 4% PFA. Spinal cords, brains, and all internal organs were properly post-fixed and dehydrated with 10–35% sucrose solution prior to embedding in OCT compound (Sakura Finetek USA Inc., Torrance, CA, USA). Per our lab’s recipe [5,12], spinal cords were transversely sectioned into 20 μm thick slices, and serial slices 100 μm apart from each other (5 sections/slide) were stained with hematoxylin-eosin (H&E). Images were taken under the microscope at 50× magnification and reconstructed via 3D Doctor (Able Software Corp, Lexington, MA, USA) for an overview of the C6-ISCG tumor mass, tumor-host interface zone, and DiI+ donor F3.hNSC distribution. 

IHC assay was carried out on one cross-section out of every 100 μm tissue that was within a 500 μm range rostral and caudal to the tumor midline. Succinctly, tissue sections were washed with PBS containing Triton X-100 (0.3%) and incubated in RT with 5% (vol/vol) normal donkey serum (Jackson ImmunoResearch Inc., West Grove, PA, USA) for 30 min before an overnight incubation with primary antibodies in 4 °C. The following primary antibodies were used individually or in varied combinations: caspase-3 (Cell Signaling Technology), neurofilament H (Thermo Fisher Scientific, Waltham, MA, USA), glial fibrillary acidic protein (GFAP; Sigma-Aldrich), NG2 proteoglycan (EMD Millipore, Danvers, MA, USA), GAP-43 (Novus Biologicals, Centennial, CO, USA), MBP (EMD Millipore), vGlut1 (Abcam, Waltham, MA, USA), CD68 (Abcam), CD86 (Novus), arginase (Santa Cruz Biotech, Santa Cruz, CA, USA), and Ki67 (Santa Cruz Biotech). Incubations with corresponding secondary antibodies (Jackson ImmunoResearch Laboratories Inc.) were performed after sufficient PBS washing. Proper antibody dilution was achieved by refining the recipes and specifications provided by the manufacturers or based on our own protocols [5,6,12,22]. Slides were coverslipped after applying an antifade mounting medium containing DAPI (VectaShield; Vector Labs) for imaging.

### 2.11. Image Analysis

Morphological examination and cell imaging were performed with a Nikon Eclipse TE300 microscope equipped with a Spot RT-Slider CCD camera (Diagnostic Instruments). Fluorescent image analysis was done using a Zeiss Axiovert 200 microscope equipped with an Axiocam CCD camera (Zeiss). All confocal images were analyzed via a Zeiss LSM1 confocal microscope equipped with Zeiss Zen microscopy software (Zen 3.10; Carl Zeiss Microimaging), with appropriate filters for FITC, Texas Red, DAPI, etc. In general, z-stack was composed of 10 images at a z-distance of 1 μm (Zeiss Zen). Each molecular marker’s fluorescent threshold range was obtained through averaging the pixel brightness of the weakest positive labeling signals and that of the strongest signals against the average background luminance level [22]. Quantification of positive IHC pixels for each marker was performed with ImageJ2^®^ to generate the percentage of the area that contained qualified signals of each antigen—i.e., immunoreactivity level—against the visual field comparably selected for all samples.

### 2.12. Statistical Analysis

Unless otherwise specified, survival data were analyzed using the rank test and the test based on medians. Behavioral, cellular, and morphological data comparison and pixel semi-quantification for histopathological, ICC, and IHC outcomes among study groups were performed with repeated-measures ANOVA or one-way ANOVA, followed with post hoc Tukey’s HSD (honest significant differences) test. Data difference between two study groups was assessed with the Student’s *t*-test. For this study, statistical significance was set at *p* < 0.05. Lastly, all statistics were computed with SPSS software version 27 (IBM Corp, Armonk, NY, USA).

## 3. Results

### 3.1. Expressions of VEGF and BDNF by F3.CD-TK and F3.CD Cells In Vitro 

**Figure 1:**
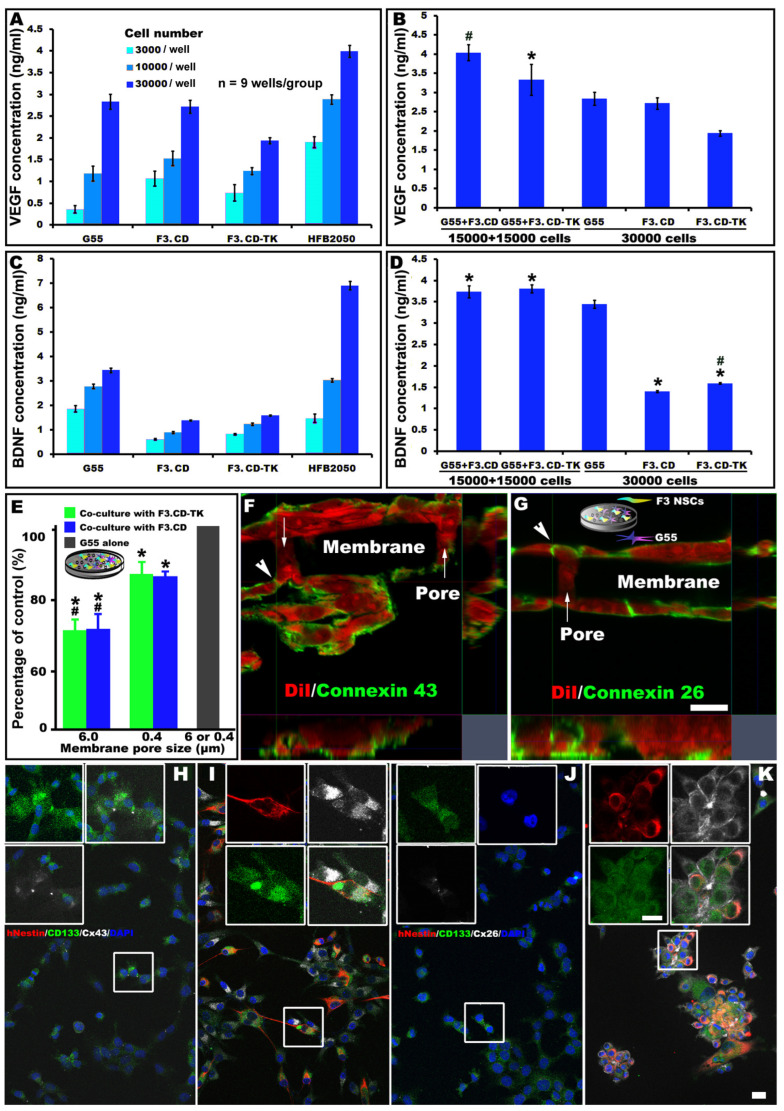
In vitro evaluations of F3.hNSC and G55 cells for key biofactors related to NSC functional multipotency and GDEPT. (**A**) All cell lines showed a dose-dependent increase in VEGF production when incubated separately. F3.CD-TK cells had significantly lower VEGF secretion than F3.CD, G55, and HFB2050 in the largest culturing concentration (*p* < 0.05, one-way ANOVA with Tukey’s post hoc test; n = 9 wells/group). (**B**) Under a 1:1 ratio, the F3.CD-TK|G55 group expressed VEGF at a mean level that was statistically similar to that of G55 alone and significantly lower than F3.CD|G55, which had an average VEGF level significantly higher than the G55-only group (*p* < 0.05: * vs. G55+F3.CD or F3.CD-TK, # vs. all other groups; two-way ANOVA with Tukey’s post hoc test; n = 9 wells/group). (**C**,**D**) ELISA of BDNF production revealed that when cultured separately, F3.CD-TK and F3.CD both had a cell dose-dependent elevation of BDNF, but at the highest cell dose, F3.CD-TK had a significantly higher mean BDNF level than F3.CD. Notably, HFB2050 prototype hNSCs as positive control cells produced the highest amount of BDNF. Coculture of F3.CD-TK or F3.CD with G55 produced BDNF that, on average, was significantly higher than that of the F3.CD-TK, F3.CD, or G55 alone group (*p* < 0.05: * vs. G55, # vs. F3.CD; two-way ANOVA with Tukey’s post hoc test; n = 9 wells/group). (**E**) Culturing G55 cells alone for 96 h produced abundant Cx43 (grey bar) in either type of the 2-chamber system. Relative to the IRL of Cx43 in either pore size monoculture set as the reference value (100%), coculture of G55 with F3.CD-TK or F3.CD cells generated comparable group average IRLs of Cx43, which were, however, lower than G55 monoculture. Cocultured cells separated by smaller pores (ϕ = 0.4 µm) had significantly higher mean IRL values of Cx43 compared to that of the larger pore size (ϕ = 6 µm) group (*p* < 0.05: * vs. G55, # vs. 0.4 µm/ϕ group; one-way ANOVA with Tukey’s post hoc test; n = 9 wells/group). (**F**,**G**) Cocultured G55 and F3.CD-TK exhibited IR of Cx43 (green) alongside the membrane of the DiI+/red cells including loci within the pores (arrows; ϕ = 6 µm; **F**). The general IRL of Cx43 appeared to be stronger than that of Cx26, another gap junction protein expressed in the cell membrane (green IR in **G**) (arrowhead: loci in the pores; ϕ = 6 µm), which was assessed separately (scale bar: 10 µm). (**H**–**K**) Compared to the 96 h cell culture data, monoculture of G55 for 24 h had very low expression of Cx43 (insets in **H**): confocal z-stacks) and Cx26 (insets in **J**): confocal z-stacks). Both Cx43 and Cx26 expressions were markedly increased between G55 and F3.CD-TK cells after 24 h coculturing (Cx43: insets in **I**; Cx26: insets in **K**; scale bars for **H**–**K** insets: 20 µm). F3.CD-TK cells (with high hNestin IRL: red) also formed Cx43- or Cx26-containing gap junctions (color code: white) with G55 cells that were CD133+ (green; i.e., cells showing yellowish overlapping pixels in **I**,**K**; scale bars: 25 µm/**H**–**K**).

The bio-factors underlying the functional multipotency (i.e., the capacity of NSCs to perform chemotaxis towards GB or neurolesion: e.g., SDF-1/CXCR4-induced migration; produce angiogenic/neurotrophic/tropic factors; form gap junctions; etc. to rebuild homeostasis) [28,29,30] of F3.CD-TK and F3.CD were evaluated as possible determinants of their antitumor and neurorepair effects (see the graphical abstract for a schematic summary). ELISA assays measured the secretion quantities of VEGF, an angiogenic/tumoritropic factor, in the medium where F3.CD-TK and F3.CD were cultured alone or with G55 human GB cells in varied mixture ratios for 96 h, to compare to G55 and HFB2050, a prototype hNSC line [29]. There were cell dose-dependent augmentations of VEGF expression in all cell lines when incubated separately (Figure 1A). However, in the largest cell concentration setting (30,000 cells/well), F3.CD-TK had significantly lower VEGF secretion than F3.CD, G55, and HFB2050 (*p* < 0.05, one-way ANOVA with Tukey’s post hoc test; n = 9 wells/group; Figure 1A). When cocultured with G55 (ratio: 1:1; total: 30,000 cells/well), the F3.CD-TK|G55 group expressed VEGF at a mean level (Figure 1B: left panel) that was statistically indiscernible from that of G55 alone (Figure 1B: right panel) and significantly lower than F3.CD|G55, which had an average VEGF level significantly higher than the G55-only group (*p* < 0.05; Figure 1B: left panel).

To examine if F3.hNSCs may promote neural repair partly through expressing BDNF, a neurotrophic factor [29,30], ELISA measurement of BDNF was performed. In cultures comprising of only a single cell type, either F3.CD-TK or F3.CD showed a cell-dose-dependent elevation of BDNF (Figure 1C). In the highest cell dose (30,000 cells/well) setting, F3.CD-TK had a significantly higher mean BDNF level than F3.CD (Figure 1D: right panel). Notably, the highest dose of HFB2050 produced markedly more BDNF than all other cell types (Fig. 1C: last panel), which, as the positive control data, validated the ELISA assay performance [29]. Coculture of F3.CD-TK or F3.CD with G55 produced BDNF that, on average, was significantly higher than that of the F3.CD-TK, F3.CD, or G55 alone group (*p* < 0.05; n = 9/group; Figure 1D). Thus, F3.CD-TK displayed a coculture profile (i.e., lower VEGF and higher BDNF) that could be more effective in concomitantly tracking/controlling tumor cells and enhancing neural recovery [13,28,31].

### 3.2. Connexins Expression and Gap Junction Formation by F3.CD-TK, F3.CD, and G55 Cells In Vitro 

One of the functional multipotency-derived abilities of NSCs is to form gap junctions with adjacent cells [30], which allows NSCs to function as optimal gene/drug delivery vehicles for gene-directed enzyme prodrug therapy (GDEPT) against GB [5,6,7,8]. Relatedly, the “bystander” tumoricidal effect results mainly from intercellular transportation of charged GCV-TP and/or ionized 5FU between F3.CD-TK (or F3.CD) and tumor cells through gap junction intercellular communication (GJIC) [32,33]. Therefore, the expression level of connexin 43 (Cx43), a primary GJIC protein, was assessed in F3.CD-TK, F3.CD, and G55 cultures using a 2-chamber system that was separated by a permeable membrane (pore ϕ: 6 µm or 0.4 µm). Culturing G55 cells alone for 96 h produced abundant Cx43 (Figure 1E: grey bar; immunostains in Figure 1F) in both types of the 2-chamber system. This immunoreactivity level (IRL) of Cx43 was set as the reference value (i.e., 100%) to accordingly calculate relative Cx43 expression amplitude in each type of cells in mixed cultures separated by the large or small pores. Coculture of G55 cells with F3.CD-TK or F3.CD cells generated comparable group average IRLs of Cx43 (Figure 1E), which were, however, lower than that of G55 monoculture. Importantly, cocultured cells separated by smaller pores (ϕ = 0.4 µm) had a significantly higher group mean IRL of Cx43 (i.e., ~90% of the G55 only group) compared to that of the larger pore size (ϕ = 6 µm) group (i.e., ~70%, n = 9 wells/group; *p* < 0.05, one-way ANOVA with Tukey’s post hoc test; Fig. 1E). Lastly, a qualitative check found that cocultured G55 and F3.CD-TK exhibited a higher general IRL of Cx43 (Fig. 1F) relative to that of Cx26, another gap junction protein (Figure 1G).

Based on stronger tumoricidal efficacy data of F3.CD-TK [6,7], the influence of G55 and F3.CD-TK coculturing on the time course of Cx43 and Cx26 expression was evaluated and compared to the G55 monoculture. Relative to the 96 h cell culture data, monoculture of G55 for 24 h had very low expression of Cx43 (Figure 1H) and Cx26 (Figure 1J). In contrast, Cx43 (or Cx26) expression was markedly upregulated between G55 and F3.CD-TK cells after 24 h of coculturing (Cx43: Figure 1I; Cx26: Figure 1K). F3.CD-TK cells (with high human nestin/hNestin IRL: red) also formed Cx43- or Cx26-containing gap junctions (immunostain color code: white) with G55 cells that had immunosignals for the main cancer stem cell (CSC) marker CD133 (green; i.e., cells showing yellowish overlapping pixels in Figure 1I,K) [34,35]. Thus, G55 cells and either line of F3.hNSCs networked extensively via GJIC between each other and among themselves. Moreover, compared to the G55 monoculture data (Figure 1H,J), F3.CD-TK cells accelerated Cx43 and Cx26 production in the cocultured cell populations (Figure 1I,K), rendering early delivery of GCV and 5FC (i.e., ≥24 h after F3.CD-TK cell administration; see below) an effective option. Lastly, F3.CD-TK cells established GJIC with CD133+ CSCs/tumor survival cells, which are a crucial target for GBM management [35].

### 3.3. A stronger Cytotoxic Effect of F3.CD-TK (vs. F3.CD) Regimen on G55 Cells and Its Dependence on Gap Junction Channels

**Figure 2:**
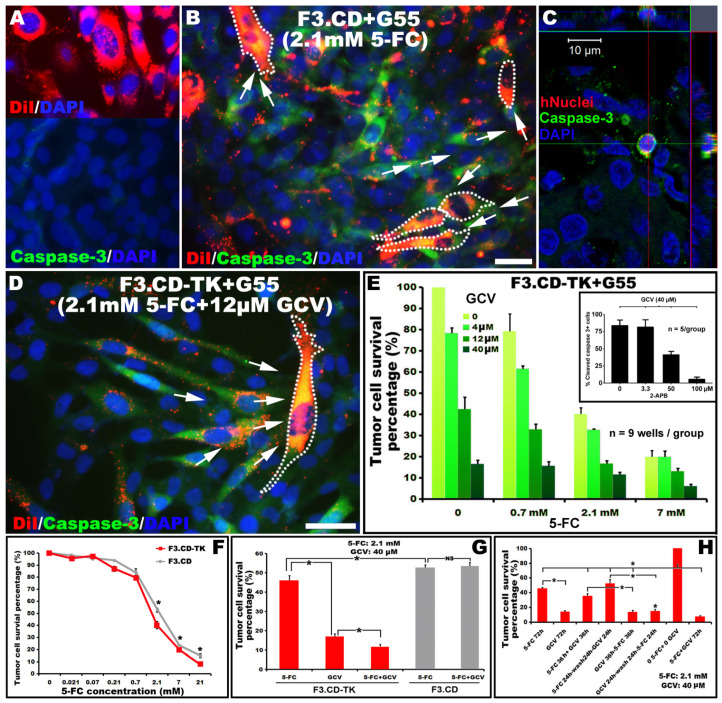
In vitro assessments of the oncolytic effect of F3.hNSC treatment against G55 cells. (**A**) Three days after adding 5FC (2.1 mM) to F3.CD (DiI+, upper panel) and G55 coculture, G55 cells (DiI−) were physically attached, although ~60% of the cells had immunostains for cleaved caspase-3, a sign of apoptosis (lower panel); (**B**), DiI+ F3.CD cells surrounded by caspase-3+ G55 cells). (**C**) Orthogonal optical slicing confirmed the proper IHC signal loci of hNuclei (red) and capspase-3 (green) in z-stack images of G55 cells (DiI−). (**D**) In contrast, 3 days after delivering 5-FC (2.1 mM) and GCV (12 µM), F3.CD-TK+G55 coculture had a large fraction (~60–70%) of G55 cells that had died and fallen off, with most residual attached G55 cells being cleaved caspase-3+. F3.CD-TK cells spread more widely (in **D**) than F3.CD cells that often appeared in small clusters (in **B**) in contact with G55 cells (**B**,**D**: 30 µm/scale bar; arrows, putative G55 chemoattractant diffusion direction to induce F3.hNSC migration). (**E**) F3.CD-TK regimen treatment dose-dependently killed G55 cells. GCV was more potent than 5FC when given individually, and 5FC+GCV was markedly more effective than 5FC or GCV administered alone. Treatment of 2-APB, a connexin channel antagonist, dose-dependently blocked the G55 cytotoxic effect of 40 µM GCV (inset in **E**), suggesting that the effect is GJIC dependent. (**F**) Regarding the oncolytic efficacy difference, F3.CD-TK cells were significantly more potent than F3.CD cells when only 5FC was given for 72 h. (**G**) F3.CD-TK had a significantly stronger oncolytic effect compared to F3.CD when 5FC and GCV were both applied for 72 h. (**H**) A similar degree of efficacy difference was observed in the F3.CD-TK+G55 coculture treated with the same prodrug dosages for 36 h, and the formula of 5FC+GCV exposure for 72 h had the strongest oncolytic effect (n = 9 wells/group; * *p* < 0.05, one-way ANOVA with post hoc unpaired Student’s *t*-test).

To revalidate the oncolytic potency of F3.CD-TK cells (prelabeled with DiI; Figure 2A: upper panel), an in vitro assay was performed as previously described [6]. In the absence of the prodrugs, F3.CD-TK or F3.CD and G55 cells normally reached ≥90% confluency in 96 h when cultured alone or cocultured in a 1:2 ratio (both settings have the same starting cell number), suggesting no growth interference between F3.hNSCs and G55 cells. Three days post-adding 5FC (2.1 mM; note: no GCV was given because F3.CD cells cannot convert it to GCV-TP and 12 µM GCV shows no effect on cell viability) [5] to the F3.CD+G55 coculture of 24 h (total time: 96 h), G55 cells (DiI negative) remained physically attached, although ~60% of the cells exhibited immunostains for cleaved caspase-3, a sign of apoptosis (Figure 2A: lower panel; Figure 2B,C). By contrast, after delivering 5-FC (2.1 mM) and GCV (12 µM), the F3.CD-TK+G55 group displayed a large fraction (~60–70%) of G55 cells that had died and fallen off, with residual attached G55 cells mostly (>90%) containing cleaved caspase-3 (Figure 2D). Furthermore, compared to F3.CD cells (Figure 2B), F3.CD-TK cells displayed a stronger tumoritropic property as they tracked and interacted with more G55 cells (Figure 2D). 

The pharmacological specificity of the prodrugs was validated by the 5FC+GCV treatment dose-response data of G55 cell survival in the coculture with F3.CD-TK (Figure 2E). Regarding the efficacy difference between the two prodrugs, GCV was more potent than 5FC when given individually, and 5FC+GCV was markedly more effective than 5FC or GCV administered alone (Figure 2E). As examples, 40 µM GCV was significantly more efficacious than 2.1 mM 5FC in causing G55 death (survival rate: 16.64 ± 1.84%/GCV vs. 45.76 ± 2.88%/5FC; *p* < 0.05; n = 5/each; repeated measures ANOVA with Tukey’s post hoc test). The combinatorial treatment of 2.1 mM 5FC and 40 µM GCV for 72 h in the F3.CD-TK+G55 setting further decreased G55 cell survival to 11.65 ± 1.24% (*p* < 0.05 vs. GCV or 5FC alone). Notably, in this setting, pre- and continued treatment of 2-aminoethoxydiphenyl borate (2-APB), an antagonist of gap junction channel subtypes (e.g., IC_50_ of 3.0 µM and 51.6 µM for Cx36 and Cx43 channels, respectively—see Discussion), dose-dependently blocked the G55 cytotoxic effect of 40 µM GCV (e.g., 50 µM of 2-APB treatment reduced G55 apoptosis by ~50%; Figure 2E inset; n = 5/group). The data suggested that GCV-TP, which was converted from GCV and released to G55 by F3.CD-TK cells, had significantly higher tumoricidal efficacy than 5FU, and this effect was GJIC-dependent (Cx43 in particular: see IC_50_ values of 2-APB above).

To compare the oncolytic potency difference between F3.CD and F3.CD-TK cells, 5FC alone was administered in doses of 2.1, 7, and 21 mM, and the result showed that F3.CD-TK was significantly more potent than F3.CD cells (n = 9 wells/group; *p* < 0.05, one-way ANOVA with post hoc unpaired Student’s *t*-test; Figure 2F). F3.CD-TK cells also had a significantly stronger oncolytic effect compared to F3.CD cells when 5FC and GCV were both applied for 72 h in the cocultured cells, eliminating ~40% more G55 cells than F3.CD cells (G55 survival rate: 11.65%/F3.CD-TK vs. 52.23%/F3.CD; Figure 2G). The data showed that GCV exerted no effect in the F3.CD+G55 coculture due to lack of GCV-TP conversion. A similar degree of efficacy difference existed in the F3.CD-TK +G55 coculture treated with the same prodrug dosages for 36 h (Figure 2H). Importantly, G55 survival rate (i.e., 11.65 ± 1.24%) was significantly lower in the 72 h 5FC+GCV exposure setting than any of the other formulas (Figure 2H).

Lastly, relative to the control group receiving neither prodrug, a sequential treatment of 5FC (2.1 mM) for 24 h followed by a 24 h washout + GCV (40 µM) for another 24 h, or GCV (40 µM) for 24 h followed with a 24 h washout + 5FC (2.1 mM) for an additional 24 h, resulted in 51.93± 3.31% and 14.32± 1.33% G55 survival rate, respectively (n = 9/group; *p* < 0.05, one-way ANOVA with Tukey’s post hoc test; Figure 2H). This outcome reconfirmed the necessity of applying 5FC and GCV simultaneously to attain the highest efficacy. Overall, the F3.CD-TK regimen was much more effective than the F3.CD formula in vitro.

### 3.4. F3.CD-TK Regimen Robustly Improved Hindlimb Locomotion and Overall Survival of C6-ISCG Animals

**Figure 3:**
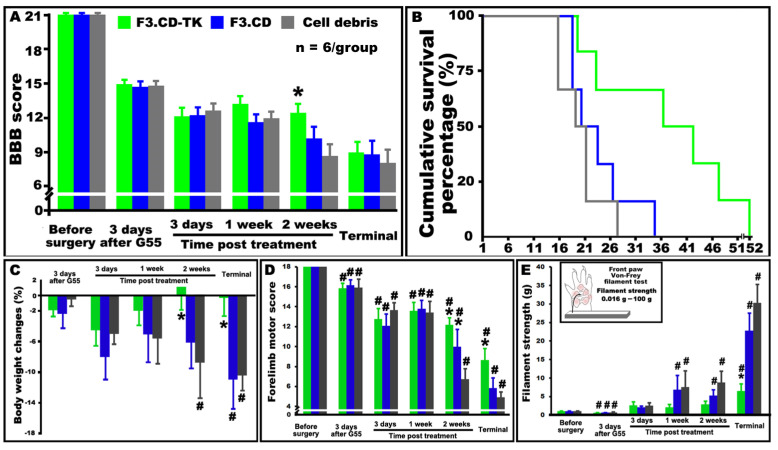
The effect of F3.CD-TK regimen on the motosensory function and overall survival of C6-ISCG animals. (**A**) C6-ISCG growth aggressively triggered the onset of the BBB score of 9 (i.e., the primary criterion of animal termination) as early as 16 days after G55 injection in the control animals that received F3.CD-TK+F3.CD cell debris+5FC&GCV. Rats treated with the F3.CD-TK regimen had significantly reduced hindlimb deficits starting ~2 weeks following the treatment compared to the F3.CD formula or control cell debris group (* *p* < 0.05, n = 6/group; two-way repeated measures ANOVA with Tukey’s post hoc test). (**B**) Kaplan–Meier curve data showed that overall survival in the F3.CD-TK-treated group was significantly longer than in the other two groups (two-sided *p* = 0.01, rank test and the test based on medians). The F3.CD formula did not significantly increase the survival of C6-ISCG rats relative to the control group (two-sided *p* > 0.05, the rank test and the test based on medians). (**C**) The F3.CD-TK regimen significantly improved mean bodyweight compared to F3.CD and cell debris groups (* *p* < 0.05 vs. F3.CD and cell debris, #*p* < 0.05 vs. 3 d after G55; two-way repeated measures ANOVA with post hoc Tukey’s test). (**D**) Animals on the F3.CD-TK regimen had significantly better forelimb locomotor function relative to the other two groups (*p* < 0.05: # vs. before surgery, * vs. cell debris or F3.CD; two-way repeated measures ANOVA with post hoc Tukey’s test). (**E**) Von Frey filament test revealed that rats in all three groups had a transient early phase mechanical hypersensitivity/allodynia, which was followed by a gradual development of mechanical hyposensitivity in the forepaw. The F3.CD-TK-treated animals had no discernible forepaw sensory abnormality for ~2 weeks of prodrug dosing, in contrast to the significantly perturbated sensory function shown in the other two groups. F3.CD-TK-treated animals had significantly less forepaw mechanical hyposensitivity than the other two groups during the terminal stage (*p* < 0.05: # vs. before surgery, * vs. F3.CD or cell debris; two-way repeated measures ANOVA with post hoc Tukey’s test).

Female adult Rowett Nude rats were re-anesthetized 7 days after C6 G55 cell implantation to receive F3.CD-TK, F3.CD, or cell debris microinjection [5]. Beginning ~48 h after F3.hNSC injection, prodrugs were given i.p. (q.d. ×5/each repeat; 5FC: 500 mg/kg/5 mL/day; GCV: 25 mg/kg/1 mL/day). Consequent to tumor growth, all animals with C6-ISCG manifested progressive hindlimb impairment. The BBB scale was used to measure hindlimb locomotor function, which, as a parametric ranking system, ranges from 0 (total paralysis) to 21 (a normal locomotion) [23]. Of note, a BBB score of 9 corresponds to the ability for a rat to make bodyweight-bearing steps, which is the essential sign of animal wellbeing. As reported before [5,6], C6-ISCG growth caused the onset of a BBB score of 9 (i.e., the primary criterion of animal termination) as early as 16 days after G55 injection in the control animals that received F3.CD-TK+F3.CD cell debris+5FC&GCV (Figure 3A). Starting ~2 weeks after prodrug treatment, rats with F3.CD-TK+5C&GCV intervention significantly reduced hindlimb deficits, compared to F3.CD (i.e., F3.CD+5FC) or control (i.e., cell debris+5FC&GCV) group (*p* < 0.05, n = 6/group; ANOVA with Tukey’s post hoc test; Figure 3A).

Kaplan–Meier curves in Figure 3B showed the effects of F3.CD-TK, F3.CD, and control treatments on the survival estimates of C6-ISCG animals (n = 6/group; termination criterion: unilateral or bilateral BBB score of ≤9). Survival in the F3.CD-TK-treated group was significantly longer than in the other two groups (two-sided *p* = 0.01, rank test and the test based on medians; Figure 3B). The F3.CD formula did not significantly increase the survival of C6-ISCG rats relative to the control group (two-sided *p* > 0.05, the rank test and the test based on medians; Figure 3B). In particular, the F3.CD-TK regimen produced a median survival time of 37 ± 4.9 days, compared to 23 ± 3.3 days and 20 ± 4.0 days, resulting from the F3.CD and control interventions, respectively. The data demonstrated that F3.CD-TK regimen delivered potent locomotor and survival improvements in a clinically relevant model of cervical ISCG.

### 3.5. F3.CD-TK Regimen Markedly Enhanced Wellbeing and Forelimb Motosensory Function of C6-ISCG Animals

To further examine how the F3.CD-TK treatment concurrently exerted local tumoricidal and pro-neurorepair impacts, the physical wellbeing and sensorimotor parameters of the C6-ISCG rats were analyzed. The F3.CD-TK regimen significantly improved group average bodyweight compared to F3.CD and control groups starting ~12 days post prodrug dosing (*p* < 0.05, two-way repeated measures ANOVA with post hoc Tukey’s test; Figure 3C). Using a modified scoring system that ranges from 0–18 (see Table S1 for details), the forelimb locomotor function was evaluated. Animals with the F3.CD-TK treatment had significantly better forelimb scores relative to those receiving the F3.CD or control intervention, beginning 12 days following prodrug administration (*p* < 0.05, two-way repeated measures ANOVA with post hoc Tukey’s test; Figure 3D). Notably, in the termination week (i.e., the terminal stage), when animals of all groups scored ≤9 on the BBB scale for hindlimb locomotion despite a marked difference (i.e., 14–17 days) in survival time, there were still significant differences in the forelimb performance between the groups, with F3.CD-TK-treated animals showing a distinctively higher mean score (i.e., ~9 vs. 5.5/F3.CD or 4.5/control; Figure 3D). The data suggest that F3.CD-TK cells might have benefited the cervical neurocircuitry through neuroprotective and neurotrophic support (e.g., BDNF) in addition to reducing the tumor burden [13,36]. 

With regards to the sensory impairment to mechanical input that is commonly associated with clinical ISCG [37], the Von Frey filament test (VFT) revealed that rats in all three groups had a transient early phase (i.e., 3 days after G55 seeding and before F3.hNSC injection) of mechanical hypersensitivity/allodynia, which was followed by a gradual development of mechanical hyposensitivity in the forepaw (Figure 3E). F3.CD-TK-treated animals had no discernible forepaw sensory change until 2 weeks after intervention compared to pre-tumor baseline function relative to the significantly perturbated sensory test scores observed in both weeks for F3.CD and control animals (Figure 3E). Animals treated with F3.CD-TK regimen also exhibited significantly less forepaw mechanical hyposensitivity than the other two groups during the terminal stage (*p* < 0.05, two-way repeated measures ANOVA with post hoc Tukey’s test; Figure 3E).

### 3.6. Autonomic Improvements in C6-ISCG Animals following the F3.CD-TK Therapy 

**Figure 4:**
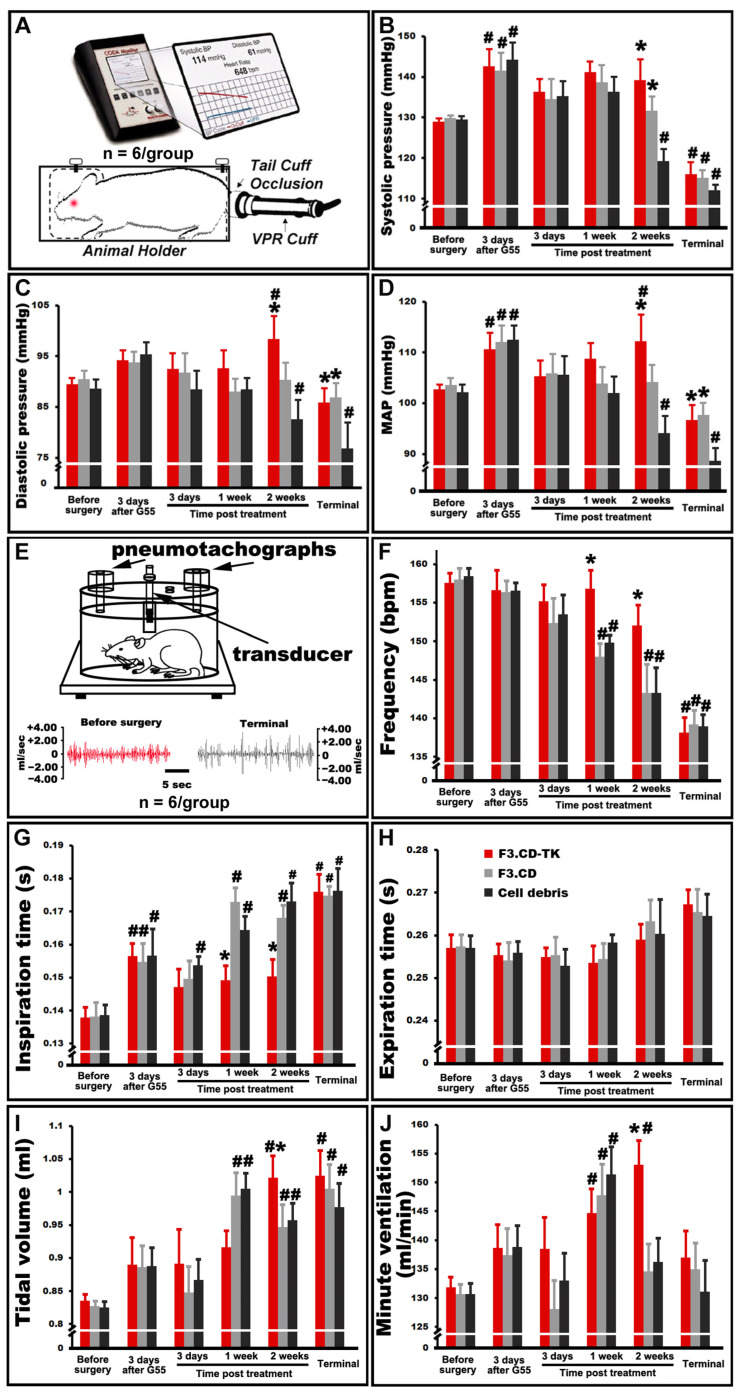
The effect of F3.CD-TK regimen on the autonomic function of C6-ISCG rats. (**A**) A noninvasive blood pressure monitoring system was used (VPR: volume pressure recording). (**B**,**C**) Three days succeeding C6 injection of G55 cells, all animals had significantly elevated systolic blood pressure, which returned to the pre-tumor level 3–7 days after F3.hNSC administration. Three weeks following C6 tumor seeding, the control group receiving cell debris had significant systolic and diastolic blood pressure decreases compared to baseline values and those of the two treated groups. (**D**) The changes resulted in significant reductions in mean artery pressure (MAP), which were efficaciously corrected by the F3.CD-TK and F3.CD treatments (*p* < 0.05: # vs. before surgery, * vs. cell debris or F3.CD; two-way repeated measures ANOVA with post hoc Tukey’s test). (**E**) Plethysmographic recording of conscious and free moving animals (upper panel) showed that in the termination week, the respiratory flow (RF: mL/s) pattern of the control rats was evidently abnormal (lower panel). (**F**) Additionally, the cell debris or F3.CD groups had significantly decreased respiratory rates (*f*: breaths/min) in weeks 1 and 2 after receiving intervention compared to pre-tumor baseline values. F3.CD-TK regimen, not F3.CD formula, maintained *f* within a normal range during that same period. (**G**,**H**) The reduced respiratory rate was caused by a significant increase of mean inspiration time (Ti: s) in the control group (**G**), while the mean expiratory time (Te: s) was comparable between the 3 groups (**H**). (**I**,**J**) The F3.CD-TK-treated group demonstrated significantly improved tidal volume (Vt: mL/per breath) (**I**) and minute ventilation (Ve: mL/min) (**J**) relative to the other two groups. Finally, no significant differences in the groups’ mean Vt and Ve were found between the 3 groups at the terminal stage (*p* < 0.05: # vs. before surgery, * vs. cell debris or F3.CD; two-way repeated measures ANOVA with post hoc Tukey’s test).

We previously reported that rats with C6-ISCG manifested progressive abnormalities in autonomic function (e.g., blood pressure and respiratory pattern changes) similar to those of clinical cervical spinal tumor cases [5]. Here, blood pressure monitoring (Figure 4A) uncovered that 3 days after C6 injection of G55 cells, all animals had significantly elevated systolic blood pressure, which returned to values statistically indistinguishable from pre-tumor levels between 3 days and 1 week after F3.hNSC administration (Figure 4B). Noticeably, in the second week following F3.hNSC or cell debris implantation (i.e., week 3 of tumor growth), control rats receiving cell debris experienced both systolic and diastolic blood pressure decreases (Figure 4B,C) compared to the baseline value, resulting in significant reductions in mean artery pressure (MAP; Figure 4D). This MAP deficit was efficaciously corrected by the F3.CD-TK regimen in the second treatment week and in the terminal stage (*p* < 0.05; two-way repeated measures ANOVA with post hoc Tukey’s test). Both F3.CD-TK and F3.CD groups had mean MAP levels that were statistically indiscernible from the baseline range and significantly higher than that of the cell debris control group (Figure 4D). 

Respiratory abnormality is a frequent underpinning of morbidity, mortality, and surgery complication for cervical spinal cord lesions, including neoplasms [5,26,38]. In this model, plethysmographic recording of conscious and free-moving animals (Figure 4E: upper panel) showed that in the termination week, the respiratory flow (RF: mL/s) pattern of the control rats was evidently abnormal (Figure 4E: lower panel). In addition, cell debris and F3.CD groups had significantly decreased respiratory rates (*f*: breaths/min) in weeks 1 and 2 after receiving the intervention compared to the pre-tumor baseline value (Figure 4F). F3.CD-TK regimen was able to maintain *f* within a normal range during that same period (Figure 4F). The reduced respiratory rate was triggered by a significant increase in mean inspiration time (Ti: s) in the control rats (Figure 4G), while the average expiratory time (Te: s) remained comparable between the three groups (Figure 4H). Importantly, in the second week following F3.hNSC injection, the F3.CD-TK-treated group demonstrated significantly better mean amplitudes of tidal volume (Vt: mL/per breath; Figure 4I) and minute ventilation (Ve: mL/min; Figure 4J) relative to the other groups (*p* < 0.05; two-way repeated measures ANOVA with post hoc Tukey’s test). However, no significant differences in the groups’ mean Vt (Figure 4I) and Ve (Figure 4J) were found between the three groups at the terminal stage, suggesting that the rat termination criteria based on a BBB score of ≤9 were justified by the correlated deterioration of respiration, which is a key vital sign.

### 3.7. The Robust Effect of F3.CD-TK Regimen on Tumor Growth in C6-ISCG Animals

**Figure 5:**
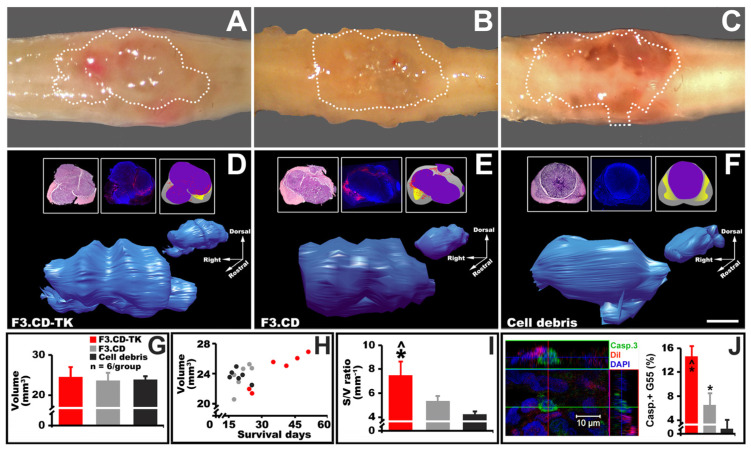
The effect of F3.CD-TK regimen on the volume, histopathological feature, and apoptosis of C6-ISCG. (**A**–**C**) Termination time gross pathology photos showed that all spinal cords had intramedullary GBs in similar sizes (i.e., the dotted areas). (**D**–**F**) A similar outcome was found in transverse histopathology images (insets: left/H&E stain, middle/fluorescent confocal z-stack, and right/camera lucida image). The F3.CD-TK-treated tissue had a much wider intratumor distribution of DiI+ F3.CD-TK cells (middle inset: F3.CD-TK/**D** vs. F3.CD/**E** and cell debris/**F** as negative control). Regarding tumor topology, relative to F3.CD- or cell debris-treated tumors, post-F3.CD-TK regimen tumors had much denser and deeper longitudinal grooves and circumferential indentations, which increased the overall surface terrain (insets: **D** vs. **E**,**F**) to produce the largest surface area (S) to volume (V) ratio (see below). (**G**) Quantification of 3D tumor reconstruction data (samples in **D**–**F**) demonstrated that the mean GB volumes in the terminal stage were statistically indiscernible between the three groups. (**H**) The F3.CD-TK regimen more effectively impeded tumor growth, as per data in the scatterplot. (**I**) The mean S/V ratio of the F3.CD-TK-treated GBs was significantly higher than the other two groups (*p* < 0.05: * vs. cell debris, ∧ vs. F3.CD; one-way ANOVA with Tukey’s post hoc test). (**J**) The average percentage of G55 cell apoptosis in F3.CD-TK and F3.CD groups was significantly higher than that of the cell debris group: F3.CD-TK-treated animals had the highest rate of activated caspase 3+ G55 cells (confocal images in the left panel; statistics in the right panel; *p* < 0.05; one-way ANOVA with Tukey’s post hoc test; n = 6 rats/group). Scale bar: 1 mm/**A**–**F**.

In this study, survival was defined according to a predetermined cutoff for hindlimb locomotor ability (i.e., a BBB scale of ≤9). Thereby, a neurologic score was used as a surrogate for tumor burden and disease progression. It is hence necessary to validate a proper correlation between the final tumor size and group mean BBB score, as well as to further specify how F3.hNSC treatment may have directly affected tumor growth parameters. Pathological and three-dimensional (3D) histopathology images showed that by the termination time, all spinal cords had intramedullary GBs of similar sizes (Figure 5A–F; quantification data in Figure 5G), although animals of the three study groups had significantly different mean survival times (e.g., the longest survival time was 52 days, 35 days, and 27 days for animals treated with F3.CD-TK, F3.CD, and cell debris, respectively; Figure 3B). 

The data in Figure 5G validates the use of the hindlimb BBB score to estimate C6-ISCG burden and overall survival. Furthermore, the F3.CD-TK regimen indeed more effectively impeded intramedullary tumor growth (via oncolysis) and host tumor tolerance (via neurorepair), which is suggested by the scatterplot exhibiting the association between tumor size and survival time (Figure 5H). Finally, no ectopic or metastatic growth of G55 cells was found in the CNS or internal organs by gross anatomy and histological examinations. However, lengthier investigations should be carried out before drawing any definitive conclusions.

### 3.8. The Modifying Effect of F3.CD-TK Regimen on the Histopathological Feature of C6-ISCG 

The axon/dendrite-mediated action potential propagation, a pivotal function of the spinal cord, can be compromised by both the absolute volume and topologic feature of an intramedullary tumor [5]. We hypothesized that for C6-ISCG of a similar general shape (e.g., Figure 5D–F) and essentially identical volume (Figure 5G), a tumor mass with longitudinal grooves and circumferential indentations would offer a more conducive environment for axon sparing than those with a much flatter surface (e.g., Figure 5E,F). To test this, 3D C6-ISCG reconstruction was performed via microscopic spinal cord cross sectional imaging to exhibit tumor topology and the F3.hNSC infiltration profile. Relative to tumors treated with F3.CD or cell debris, post-F3.CD-TK regimen tumors had much denser and deeper longitudinal grooves and circumferential indentations, which increased the overall surface terrain (Figure 5D vs. Figure 5E,F; insets: left/H&E stain, middle/fluorescent confocal z-stack, and right/camera lucida image generated by combining the H&E and fluorescent images) to produce the largest surface area (S) to volume (V) ratio; the S/V ratio was significantly higher than the other two groups (*p* < 0.05, one-way ANOVA with Tukey’s post hoc test; Figure 5I). These surface grooves and indentations were possibly dug up by streams of migrating F3.CD-TK cells via releasing GCV-TP and 5FU to kill G55 cells and themselves. This was suggested by the detection of DiI-prelabelled F3.CD-TK cells within the interface space between the tumor mass and the spinal cord parenchyma (i.e., DiI+ red cells and red schematic dots in the middle and right insets, respectively; Figure 5D–F). Markedly, F3.CD-TK-treated tissue possessed a much wider intratumor distribution of DiI+ F3.CD-TK cells, which, in line with aforementioned in vitro data, might have served as another underpinning for their more potent oncolytic effect when compared to F3.CD (Figure 5D/F3.CD-TK vs. Figure 5E/F3.CD and Figure 5F/cell debris as negative control).

### 3.9. The Effect of F3.CD-TK Regimen on Inducing Apoptosis of C6-ISCG Cells 

The average percentage of G55 cell apoptosis in F3.CD-TK- or F3.CD-treated animals at termination stayed significantly higher than that of the control group. The F3.CD-TK regimen group had the highest rate of apoptotic tumor cells (i.e., activated caspase 3+ G55 cells/per section; confocal images in Figure 5J, left panel): 14.9 ± 1.6/F3.CD-TK+5FC&GCV vs. 6.6 ± 1.9/F3.CD+5FC or 1.1 ± 2.1/cell debris+5FC&GCV (*p* < 0.05, one-way ANOVA with Tukey’s post hoc test; n = 6 rats/group; Figure 5J: right panel). 

### 3.10. F3.CD-TK Regimen Rescued Host Neurites within the Interface Space between the Tumor and Spinal Cord

**Figure 6:**
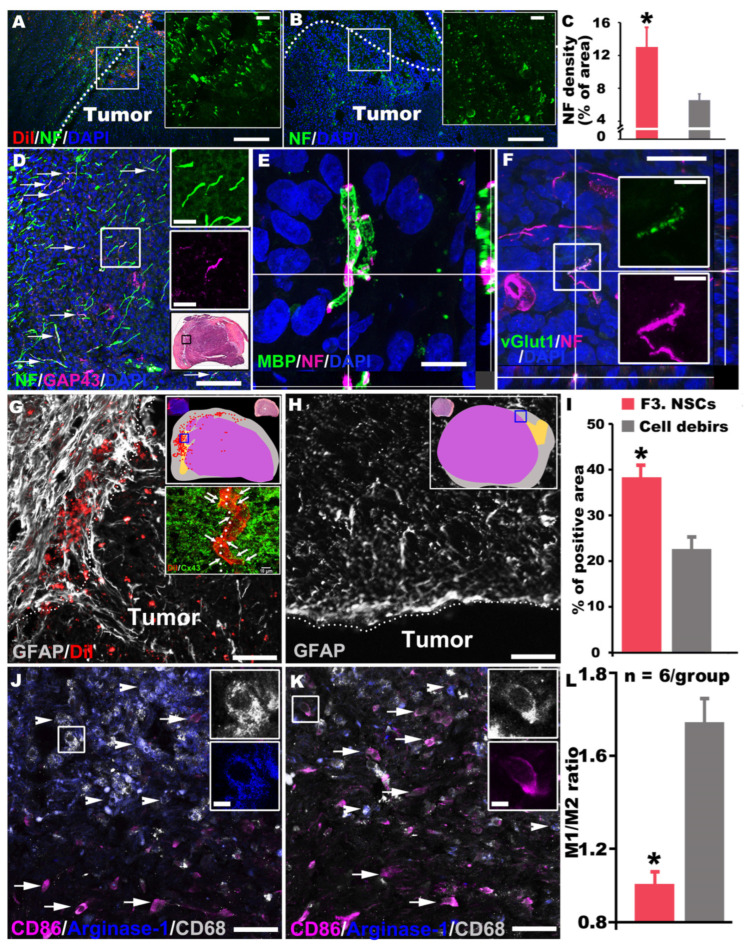
The impact of F3.CD-TK regimen on host neurite rescuing as well as neuroinflammation and neuroimmune modulation. (**A**–**C**) There was a significantly augmented group average IRL of neurofilament H (NF-H) in the host neurites (DiI−) of F3.CD-TK-treated C7 dorsolateral spinal cords within the grooved/indented interface around Rexed lamina-I (RL-I) compared to cell debris controls (n = 6/group; * *p* < 0.01, Student’s *t*-test). (**D**) About 10–15% NF-H+ (upper inset) neurites expressed the growth-associated protein 43-kD (GAP-43) that is a marker of neurite growth (arrows in (**D**); bottom inset: tissue location of the images). (**E**) Confocal imaging disclosed that ~6–10% NF-H+ axons within the interface zone were ensheathed by a thin layer of myelin basic protein (MBP) in F3.CD-TK-treated tissue only. (**F**) A similar fraction (i.e., 2–10%) of the NF-H+ neurites expressed vesicular glutamate transporter 1 (vGlut 1), an instrumental molecule of the propriospinal fiber terminals. (**G**–**I**) A significantly increased GFAP IRL presented along the interface zone and mainly on the host parenchyma side (left to the dotted line; upper inset: area sampled; **G**) in F3.CD-TK-regimen-treated spinal cords relative to controls (inset: area sampled; **H**) receiving cell debris (n = 6/group; * *p* < 0.01, Student’s *t*-test; **I**). Notably, cells with augmented GFAP expression contained no DiI (**G**), indicating that they were host astrocytes. DiI+ F3.CD-TK cells in the interface zone of an adjacent tissue section (dotted line in **G** lower inset) formed Cx43 gap junctions with both host cells (left side) and G55 cells (right side), which were revealed by yellow IR signals pointed by arrows in **G** lower inset. (**J**–**L**) Triple immunostaining disclosed that the F3.CD-TK regimen significantly increased the alternatively activated (arginase+/CD68+ and anti-inflammatory/immune modulatory) M2 microglia (arrowheads, **J**) quantity but decreased classically activated (CD86+/CD68+ and pro-inflammatory/cytotoxic) M1 (arrows, **J**) numbers when compared to the cell debris control formula (M2/arrowhead; M1/arrow in **K**), reducing the M1/M2 ratio (**L**; n = 6/group; * *p* < 0.01, Student’s *t*-test). The M2 increase in F3.CD-TK-treated tissues mostly occurred at loci deeper inside the host spinal cord (i.e., the upper left section in **J**) while the M1 cells were concentrated in areas adjacent to the tumor interface (i.e., the lower right section in **J**). In contrast, M1 microglia distribution was more even in the control tissue (**K**). Scale bars: 250 µm/**A**,**B**, and insets; 125 µm/**D** and insets; 10 µm/**E**; 20 µm/**F** and insets; 100 µm/**G**,**H**; and 80 µm/**J**,**K**, and insets.

To assess whether F3.hNSCs might have mitigated ISCG-associated neuroinjuries while eliminating ISCG cells, the presence of host axons/neurites in the interface space between the tumor and neighboring neural parenchyma was examined. Compared to the control, the F3.CD-TK-treated spinal cords had significantly increased group average IRL values of neurofilament H (NF-H), a mature neuronal marker [29], in the host neurites (i.e., DiI−); these neurites ran through the grooved/indented tumor periphery that interfaced with C7 dorsolateral parenchyma, around Rexed lamina-I (RL-I; n = 6/group; *p* < 0.01, Student’s *t*-test; Figure 6A–C). F3.CD formula-treated tissues had a slightly increased mean IRL of NF-H, which was not significantly higher than that of the control group. A small proportion of the NF-H+ neurites (~10–15%) expressed the growth-associated protein 43-kD (GAP-43, a marker of axonal growth), indicating that certain degrees of neurite repair were induced or permitted by the F3.CD-TK treatment (Figure 6D). In either group, no NF-H+ axons/neurites were detected in loci 300–350 µm below the surface of the tumor core.

Confocal z-stack imaging disclosed that some NF-H+ axons (~6–10%) within the interface zone (depth on either side: 200 µm; total width: 400 µm) were ensheathed by a thin layer of myelin basic protein (MBP: a structural myelin component), suggesting that these axons had likely been repaired and/or preserved by the F3.CD-TK therapy (Figure 6E) since such signals were absent in the control tissue. Because these axons existed near the tip of the dorsal horn, they likely belonged to the local-circuit interneurons forming short and long propriospinal projections [39]. This postulation was partially confirmed by the observation that a similar fraction (i.e., 2–10%) of the NF-H+ neurites expressed vesicular glutamate transporter 1 (vGlut 1; Figure 6F), an instrumental molecule for propriospinal fiber terminals [12]. The data demonstrated that the F3.CD-TK regimen was able to preserve and repair host neurites, including those from local neurocircuits crucial to sustaining locomotion after SCI [12,13,40].

### 3.11. F3.CD-TK Treatment Improved Intraparenchymal Microenvironment via Neuroinflammation/Neuroimmune Modulation and GJIC Formation

The effects of C6-ISCG and F3.CD-TK or control treatment on local neuroinflammation was investigated by measuring the presence and severity of a tumor-surrounding glial scar, the product of reactive astrogliosis that is a neuroinflammatory process and product [41]. This was accomplished through evaluating the IRL increase of GFAP (the primary intermediate filament of astrocyte), a hallmark of reactive astrogliosis that plays important roles in restricting lesion expansion and, depending on the diversity of reactive cell types and cell action mode, hindering or facilitating GB growth vs. neural recovery [42]. A significantly elevated GFAP IRL was detected along the interface zone and predominantly on the host parenchyma side in F3.CD-TK-treated spinal cords relative to the controls (Figure 6G–I). Cells with augmented GFAP expression contained no DiI (Figure 6G upper inset: sampling area schematic), indicating that they were host astrocytes. Likewise, image analysis taking account of combinatorial factors of DiI and IR of NF-H, a neuronal marker (or NG2, an oligodendrocyte precursor marker) did not find evidence of neuronal (or oligodendrocytic) differentiation of F3.hNSCs: i.e., no NF-H+ (or NG2+) cells were DiI+ in the same tissue. DiI, a lipophilic dye, associates with pre-labeled cell membranes and does not contaminate the membranes of other cells [43]. Moreover, DiI+ F3.CD-TK cells in the interface zone (dotted line in Figure 6G and lower inset) formed Cx43 gap junctions with cells on either side, indicating that they established GJIC with both host cells and G55 cells (Figure 6G lower inset).

Because the main cellular components of the glial scar are astrocytes, microglia, and NG2 glia, which constitute an important part of the microenvironment for host-tumor interaction [42], we examined the effect of F3.CD-TK therapy on the polarization state of microglia (i.e., CNS-resident macrophages immunopositive for CD68+) [44] inside the host tissue 200–400 µm away from the interface midline via calculating the ratio of M1/M2 microglia (M1: CD86+/CD68+: pro-inflammatory and cytotoxic; M2: arginase+/CD68+: anti-inflammatory and immunomodulatory) [45]. On the basis of triple immunostaining results, the F3.CD-TK regimen significantly decreased M1 numbers but augmented M2 quantity (Figure 6J) when compared to the cell debris control formula (Figure 6K), reducing the M1/M2 ratio in the cervical spinal cord (Figure 6L). Importantly, the M2 increase in F3.CD-TK-treated tissues mostly occurred at loci deeper inside the spinal cord neuroparenchyma, further away from the host-tumor border (i.e., the upper left section in Figure 6J), while the M1 cells were concentrated in the peripheral areas adjacent to the tumor interface (i.e., the lower right section in Figure 6J). In contrast, M1 microglia distribution was more even in the control tissue (Figure 6K). The outcome suggested that the F3.CD-TK regimen modulated the neuroinflammation and neuroimmune microenvironment of the spinal cord to strengthen tumor restriction and host neural repair [34,41,46]. Taken together, the F3.CD-TK treatment more efficaciously killed G55 cells, which, acting alongside its effects on tumor topology modification, neuroinflammation/neuroimmune modulation, and neural repair, jointly stalled C6-ISCG growth and resultant neurological deficits.

### 3.12. The In Vivo Self-Clearance Rate Difference between F3.CD-TK and F3.CD Cells

**Figure 7:**
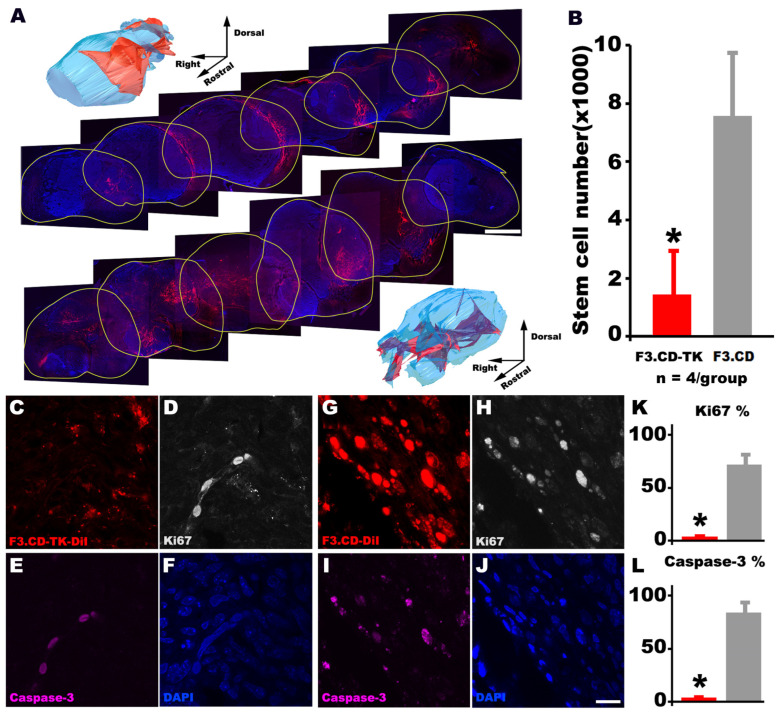
Comparison of self-clearance rate between F3.CD-TK and F3.CD cells in vivo. (**A**) The mean number of DiI+ cells was significantly less in F3.CD-TK-treated animals (upper panel) than F3.CD group (lower panel). (**B**) The residual F3.CD-TK cell number, averaged from DiI+ cell numbers from 6 slices (section thickness: 20 µm), with each sampled from one consecutive 500 μm-long tissue block, was merely ~25% of that of F3.CD-treated tissues (* *p* < 0.05, Student’s *t*-test). (**C**–**F)** Only ~5% of these residual F3.CD-TK cells (DiI+, **C**) expressed Ki67, a cell proliferation marker in the nucleus (**D**) and even fewer of which (~4%) had nuclear presence of cleaved capase-3 (**E**; DAPI nuclear stain in **F**), suggesting that they were in senescence after the vast majority had died off (statistics in **K**). (**G**–**J**) In contrast, ~72% of the residual F3.CD cells (**G**) exhibited nuclear IR of Ki67 (i.e., proliferating, **H**) and cleaved caspase-3 (i.e., experiencing apoptosis; **I**; DAPI nuclear stain in **J**; statistics in **L**). The F3.CD-TK/5FC+GCV regimen therapy more effectively eliminated proliferating donor cells. Scale bars: 700 µm/**A**; 40 µm/**C**–**J**.

For targeted gene therapies mediated by stem cells, it is pivotal to profile donor fate in vivo to improve safety due to risks of immune responses and oncogenesis. Thus, spinal cord sections were collected from representative C6-ISCG animals (i.e., showing individual BBB scores close to the group mean; n = 4/group) that were treated with the formula of either F3.CD-TK or F3.CD (see above). Examination of the tissue revealed that the mean number of DiI+ cells was significantly less in F3.CD-TK animals (Figure 7A, upper panel) compared to F3.CD group (Figure 7A, lower panel), suggesting that F3.CD-TK cells were more effectively eliminated mainly by the combined suicide cytotoxicity of 5FU and GCV-TP. Specifically, the residual F3.CD-TK cell number, averaged from DiI+ cell numbers from 6 slices (section thickness: 20 µm) with each sampled from one consecutive 500 μm long tissue block (i.e., total length: 6 × 500 µm = 3 mm; 1.5 mm rostral—1.5 mm caudal to the middle line of the tumor), was merely ~25% of that of F3.CD-treated tissues (Figure 7B; *p* < 0.05, Student’s *t*-test). In addition, only ~5% of these residual F3.CD-TK cells expressed Ki67, a cell proliferation marker (Figure 7C,D), compared to ~72% of F3.CD cells (Figure 7G,H; statistics in Figure 7K). Consequentially, even fewer residual F3.CD-TK cells (~4%) were immunoreactive for activated caspase 3 (Figure 7E,F), for which the majority (80.38 ± 7.40%) of residual F3.CD cells displayed IR (i.e., cleaved caspase 3+; Figure 7I,J; statistics in Figure 7L). The data demonstrated that in this C6-ISCG model, F3.CD-TK cells, compared to F3.CD, had significantly higher self-clearance rates in response to the formulated dosing of 5FC and GCV. This is an important favorable property of donor cells for their application in GDEPT against cancer. Lastly, the drastically reduced spinal cord presence of F3.CD-TK cells in the final phase could be mainly responsible for terminal decline of neurological function in the treated C6-ISCG animals (see Figure 4) due to diminished therapeutic effects.

## 4. Discussion

In theory, one promising strategy to control ISCG resides in establishing targeted therapies to control the infiltrative growth of ISCG while in parallel mitigating tumor-introduced neuroinjuries [3,31,47]. Indeed, conventional anticancer drugs are designed to manage tumor cells only. Thus, the overall prognosis following current chemotherapies is critically dependent on the patient’s baseline health condition, which defines the endogenous repair and functional recovery capacity following tumor mitigation (i.e., it is the host system that restores function) [3,37]. Genetically engineered hNSCs with tumoritropic ability and oncolytic function have been experimentally investigated for better tracking and elimination of brain GBM and ISCG [5,6,7,8,9]. However, whether this oncolytic strategy could concurrently repair the lesioned CNS has remained unstudied until now. This multimodal study began to address this important question with mechanistic explorations. We have uncovered that a F3.CD-TK regimen, relative to F3.CD, not only more potently controlled G55 C6-ISCG but also better promoted neurorepair to enhance function and overall survival of C6-ISCG animals. F3.CD-TK cells showed much wider tumor infiltration to trigger a larger quantity of G55 apoptosis and the ability to provide stronger neurotrophic, anti-inflammatory, and neuroimmune modulatory effects (i.e., major mechanistic leads). Compared to F3.CD, F3.CD-TK cells also had a significantly higher self-clearance rate after prodrug dosing. Collectively, our data suggests that the overall higher F3.CD-TK cell-mediated therapeutic benefit is empowered concomitantly by the dual gene-directed enzymatic prodrug conversion and the functional multipotency of hNSCs (e.g., tumoritropic migration, GJIC formation, trophic factor secretion, anti-neuroinflammation, and neuroimmune modulation; see the graphical abstract for a schematic summary of this investigation). 

ISCG growth causes compression, resulting in downstream injuries (e.g., ischemia, neuronal death, etc.), functional alterations (e.g., sensorimotor deficits, autonomic disorders, etc.), and clinical symptoms (e.g., pain, muscle weakness, paralysis, cardiorespiratory abnormalities, etc.) [1,2,3]. Cooperatively, GB cells activate cascades of pathophysiological events of glutamate release (i.e., excitotoxicity) [48]; iron homeostasis perturbation (e.g., oxidative stress) [49]; and cytokine, growth factor, and enzyme discharges [28,50]. These activities lesion host neural cells, compromise neuroimmune responses, and modify the extracellular matrix to exacerbate the cancer spreading and tissue destruction. Clinically, surgical decompression of the cervical and thoracic spinal cord can trigger reperfusion injury with severe neurological consequences [51]. Administration of common chemotherapeutics and brain radiotherapy have been associated with long-term cognitive decline, likely due to cerebral necrosis and damage to neurogenesis niches in the hippocampus, a brain region crucial for learning, memory formation, and cognition [52,53]. Hence, it is pivotal to devise a targeted GB therapy that concurrently kills cancer cells and repairs the injured CNS. 

This multifaceted therapeutic demand has accentuated the necessity of investigating NSC-based GDEPT approaches for brain tumors [7,8,9,10,11]. Our group earlier established the first clinically relevant rat cervical xenograft ISCG model and used it to study the tumoricidal effects of F3.hNSC-based GDEPT [5,6]. NSCs, which possess both phenotypic and functional multipotencies [30], have long been exploited to execute CNS repair via neural cell replacement, multifunction-mediated homeostasis, gene therapy, and drug or molecule delivery [54]. However, unlike the brain [55], the post-developmental mammalian spinal cord, intact or injured, is generally not permissive to either neuronal differentiation of NSCs or functional integration of transplanted neurons [56]. Accordingly, cell marker assays detected no neural cell differentiation in our study. The strong neurological benefits of the F3.CD-TK regimen for this model appear to be derived from the functional multipotency-produced events as adjuvant mechanisms of neural recovery together with the oncolytic effect.

Relative to F3.CD, F3.CD-TK in single or G55-combined cultures produced a significantly higher amount of BDNF, whereas their expression of VEGF was discernibly lower. BDNF is a potent neurotrophin, which, through activating TrkB receptors, can preserve neurons and synapses against excitotoxicity, oxidative stress, ionic imbalance, neuroinflammation, and other cytotoxic insults generated by intramedullary GB, neurotrauma, or neurodegeneration [12,29]. BDNF secreted by NSCs was shown to correct cognitive deficit in Alzheimer’s disease and radiation injury models [57]. By producing more BDNF, F3.CD-TK cells may possess a better capacity to enhance sensorimotor and autonomic improvements in C6-ISCG animals. In fact, the detectably weaker in vivo neurorepair benefit of the F3.CD treatment may be partly attributed to the cells’ lower BDNF production than F3.CD-TK. Apart from the angiogenic role, VEGF gradients set up by GB cells provide a chemotactic cue for NSC homing via interaction with VEGFR-1 [28]. Relative to F3.CD and G55, the significantly lower VEGF expression in F3.CD-TK cells could partially underlie their more frequent individual penetration of G55 clusters in vitro and wider infiltration inside the C6-ISCG mass in vivo to inhibit tumor growth. Future studies should validate the roles of BDNF, VEGF, and other factors (e.g., GDNF) in hNSC-mediated neurorepair for GDEPT of ISCG through conducting specific antagonism, knockdown, and knockout assays [28,30].

A prominent feature of NSC functional multipotency checked in F3.CD-TK and F3.CD cells for the first time is their ability to form gap junctions with G55 cells. This capability must be assessed before the two cell lines can be judiciously used to treat ISCG because the bystander tumoricidal effect of potently charged GCV-TP and ionized 5FU (pKa: 7.93–8.05) in the acidic cancer extracellular environment is realized through GJIC transfer to kill tumor cells. While GCV-TP directly inhibits polymerase and breaks DNA double-strand, 5FU suppresses thymidylate synthase and RNA synthesis, respectively (note: non-ionized 5FU in more physiological pH environment crosses cell membrane through diffusion and a saturable membrane transport system; see illustrations in the graphical abstract) [58,59,60]. Our assays confirmed the expression of Cx43 and Cx26, main GJIC connexins in G55 and NSCs, by all three cell lines. Of note, connection of F3.hNSCs with G55 through smaller pores (ϕ = 0.4 µm) in the separation membrane induced a significantly stronger expression of Cx43 than that of the larger pore setting (ϕ = 6 µm), permitting intercellular transfer of GCV-TP and ionized 5FU under extremely slim physical contact between F3.hNSCs and G55. The mechanism underlying this phenomenon is not yet clear. Previous work showed that Cx43 expression in cardiomyocytes could be regulated by mechanical stress ignited by ECM conditions [61], and Cx43 degradation from the cell surface was reduced by physiologically relevant types of stress [62]. It is possible that F3.hNSCs extending thinner filopodia through smaller pores experienced more mechanical and cytosolic stress than when growing inside larger pores. Importantly, the 2-APB dose-response data confirmed that the F3.CD-TK regimen’s GCV efficacy is dependent on the functionality of Cx43 GJIC since treatment of 50 µM 2-APB (Cx43 IC_50_: 51.6 µM) [63] mitigated G55 apoptosis by ~50% in vitro.

F3.CD-TK and G55 coculture expedited Cx43 and Cx26 production, which justified the initiation of GCV and 5FC delivery at ≥24 h after F3.CD-TK cell administration in vivo. A likely underpinning is that phosphorylated GCV augments astrocytic differentiation of GB cells to increase connexin synthesis [64,65]. Notably, the data of GJIC formation between F3.CD-TK and G55 cells expressing CD133, a marker of CSCs [33,34,35], indicated that gap junction-mediated intercellularly transport of GCV-TP and ionized 5FU may directly damage CSCs, a crucial target for GBM management [35]. These findings suggest that Cx43 may be a salutary player in ISCG prognosis. In fact, though Cx43 has been recognized to have a perplexing role in GBM biology, a recent meta-analysis study revealed that Cx43 expression level was positively correlated with the overall survival of GBM patients and negatively correlated with tumor grades [66]. Here, in situ, F3.CD-TK cells formed Cx43 GJIC with both G55 cells and host neural cells, which—in addition to inhibiting C6-ISCG—may have improved intraparenchymal homeostasis as reported before [67].

To determine whether F3.CD-TK cells could modulate neuroinflammation, the IRL of GFAP was measured because the reactive astrogliosis process driven by inflammatory molecules paradoxically contributes to lesion healing and GB confinement or invasion [41,68]. Treatment with F3.CD-TK regimen produced a significantly increased GFAP IRL mostly inside the host territory within the interface zone to form a stronger astroglial scar. It has been reported that both in vitro and in vivo, the tumor-enclosing astroglial scar-housed microglia, compared to those in GBM alone, released 3.2-fold more glutamate to stimulate astrocytic monoamine oxidase-B (MAO-B) activity and chondroitin sulfate proteoglycans (CSPGs) deposition to restrict GBM growth [41]. 

GB cells can augment their growth, invasion, survival, metastasis, and migration, as well as radio/chemotherapy resistance by manipulating the tumor microenvironment (TME) through, for instance, polarizing microglia [69]. However, data have been extremely sparse regarding astroglial scar-associated microglia as a forefront modulator of host-tumor neuroimmune interactions. We have here uncovered that inside the host tissue stripe of the astroglial scar, the F3.CD-TK regimen significantly decreased the level of proinflammatory M1-polarized microglia but increased that of pro-neural repair M2. This shift is expected to ameliorate locomotion recovery as previously found in SCI studies [12,46]. Conversely, the M1-like microglia in the F3.CD-TK-treated tissues were concentrated in areas immediately adjacent to the tumor, suggesting that the treatment also improved the local milieu’s anticancer potential since a typical GB TME tends to suppress the M1/M2 ratio to favor tumor invasion [70].

The higher antitumor efficacy of the F3.CD-TK regimen may also be associated with the following mechanisms. GCV, vs. 5FC, has a much lower IC_50_ value in suppressing human glioma cells. The IC_50_ for the suicide effect was 0.130 ± 0.045 µM in hMSC transfected with HSV-TK [71] compared to 36.5 μM in hMSC-CD cells [72]. Moreover, a synergistic effect of GCV-TP and 5FU has been found to be mediated by CD/5-FC-triggered dTTP and dGTP reduction via allosteric ribonucleotide reductase regulation to mitigate dGTP’s role as the endogenous competitor of GCV-TP [73]. Conversely, both GCV and its more lipophilic metabolite could interact with multidrug-resistant proteins (MRPs) in breast cancer cell lines, to inhibit the efflux of other chemotherapeutics [74]. Such an event may also occur in the F3.CD-TK and G55 settings because differential expressions of MRPs were detected in colorectal cancer patients who were resistant to 5FU treatment [75].

Our study demonstrated for the first time that the F3.CD-TK regimen has stronger oncolytic efficacy compared to the F3.CD formula for C6-ISCG and is able to reshape ISCG’s topology by generating longitudinal grooves and circumferential indentations in the tumor periphery to spare host neurites. With the simultaneous availability of NSC’s neurotrophic effect, the preserved neurites appeared to have been repaired and maintained to sustain local neurocircuit operation. Therefore, future oncological strategies should focus on jointly managing the volume and topology of ISCGs to maximize neurological recovery. Other valuable new data points were the significantly stronger somatosensory benefit and higher self-clearance rate of the F3.CD-TK cells: sensory disorders of numbness, tingling, and pain are common symptoms of clinical ISCG [37], and for safety, the removal of genetically engineered donor stem cells is crucial to mitigating mutation and tumorigenesis risks [31].

There are certain limitations in the present study. For example, although G55 cells fulfill major criteria of a patient-derived xenograft and are more invasive than other human GB lines (e.g., U87) [6], no examination was performed to characterize the evolvement of phenotypic heterogeneity in each tumor formed in situ, nor the development of chemoresistance. The ability of F3.hNSCs to track down migrating tumor cells over long distances was not examined. The coordinated migratory pattern of GB cells was not investigated to determine whether F3.CD-TK may impede any signaling process underlying this representative behavior of GB cells [76]. The investigation did not collect long-term data on the implanted F3.CD-TK cells relative to F3.CD concerning safety or efficacy issues. In the future, the dual effects of the F3.CD-TK regimen can be differentiated by comparing the efficacy between the treatments of F3.CD-TK, F3.CD, and direct intratumor expression of CD and TK via a viral (or chemical) vector. The current findings should be cross-examined in more mammalian models of cervical ISCG (e.g., patient-derived xenograft tumors) when they become available.

## 5. Conclusions

We uncovered that F3.CD-TK—dual gene-engineered hNSCs—concurrently repaired neuroinjury while tracking/eliminating ISCG cells. In vitro and in a rat C6-ISCG model, F3.CD-TK cells, which expressed CD and TK to convert nontoxic 5FC and GCV respectively into tumoricidal 5FU and GCV-TP near cancer cells, demonstrated a significantly higher degree of intratumor penetration, tumor topology modification, oncolytic efficacy, neurorecovery effect, and self-clearance when compared to F3.CD cells that produced CD only. The GCV-TP cytotoxic effect was Cx43 GJIC dependent. The F3.CD-TK regimen represents a proof-of-principle example that hNSC-based GDEPT can be both oncolytic and neurotherapeutic to treat ISCG and related neuroinjuries.

In conclusion, this investigation has shed new light on targeted GB therapy research, and the following is its most valuable: the F3.CD-TK-based GDEPT not only kills high-grade ISCG cells but also protects and repairs neuroinjury. Therefore, the hNSC-based multimodal strategy has a strong capacity to concurrently address the two most important requirements for effectively treating CNS GB. Our findings may facilitate the launch of more research initiatives about this approach and help develop clinical trials on the use of dual gene-engineered hNSCs to treat ISCG and other CNS malignant tumors.

## Data Availability

All study data are included in the article. For seeking other information and materials that are related to this project, please contact the corresponding author.

## Supplementary Materials

The following supporting information can be downloaded at: https://www.mdpi.com/article/10.3390/cells13181522/s1, Table S1: The rating sheet with scores applied for each behavioral item of the forelimb.
