# Supplementary material for: Concurrent Oncolysis and Neurolesion Repair by Dual Gene-Engineered hNSCs in an Experimental Model of Intraspinal Cord Glioblastoma
Source: Cells. 2024 Sep 11;13(18):1522. doi: 10.3390/cells13181522 (PMC11429792; doi:10.3390/cells13181522)
Supplement: Supplementary file 1 [file cells-13-01522-s001.zip › cells-3080162-supplementary.pdf]

Table S1. The rating sheet with scores applied for each behavioral item of the forelimb

Rat #:	Days post-surgery:	Date:	Scores: /L; /R; /Average

	Articular movements of the forelimb		
	Shoulder	Elbow	Wrist
Absent	0	0	0
Slight	1	1	1
Normal	2	2	2

	Weight support	
	Stationary	Active
Forelimb	1	1

	Digit position			Stepping								
	Flexed	Atonic	Extended	Paw placement at initial contact				Paw orientation during liftoff			Movement	
				Dorsal	Plantar							
					I	E	P	I	E	P	Regular	Irregular
Forelimb	0	1	2	0	1	1	2	1	1	2	2	1

	Forelimb and hindlimb coordination
Absent	0
Occasional	1
Frequent	2

Notes: (1) Abbreviations: I, internal; E, external; P, parallel; L, left forelimb; R, right forelimb. (2) These points are added up to the total score of 18 for the forelimb. (3) This sheet is adapted from Ref. [24] (Martinez, M., Brezun, J.M., Bonnier, L., Xerri, C. A new rating scale for open-field evaluation of behavioral recovery after cervical spinal cord injury in rats. Journal of neurotrauma 2009; 26, 1043-1053).
